# Frequent Social Media Use and Experiences with Bullying Victimization, Persistent Feelings of Sadness or Hopelessness, and Suicide Risk Among High School Students — Youth Risk Behavior Survey, United States, 2023

**DOI:** 10.15585/mmwr.su7304a3

**Published:** 2024-10-10

**Authors:** Emily Young, Jessica L. McCain, Melissa C. Mercado, Michael F. Ballesteros, Shamia Moore, Laima Licitis, Joi Stinson, Sherry Everett Jones, Natalie J. Wilkins

**Affiliations:** ^1^Division of Adolescent and School Health, National Center for Chronic Disease Prevention and Health Promotion, CDC, Atlanta, Georgia; ^2^Division of Violence Prevention, National Center for Injury Prevention and Control, CDC, Atlanta, Georgia; ^3^Division of Injury Prevention, National Center for Injury Prevention and Control, CDC, Atlanta, Georgia; ^4^Oak Ridge Institute for Science and Education, Oak Ridge, Tennessee

## Abstract

Social media has become a pervasive presence in everyday life, including among youths. In 2023, for the first time, CDC’s nationally representative Youth Risk Behavior Survey included an item assessing U.S. high school students’ frequency of social media use. Data from this survey were used to estimate the prevalence of frequent social media use (i.e., used social media at least several times a day) among high school students and associations between frequent social media use and experiences with bullying victimization, persistent feelings of sadness or hopelessness, and suicide risk. All prevalence estimates and measures of association used Taylor series linearization. Prevalence ratios were calculated using logistic regression with predicted marginals. Overall, 77.0% of students reported frequent social media use, with observed differences by sex, sexual identity, and racial and ethnic identity. Frequent social media use was associated with a higher prevalence of bullying victimization at school and electronically, persistent feelings of sadness or hopelessness, and some suicide risk among students (considering attempting suicide and having made a suicide plan), both overall and in stratified models. This analysis characterizes the potential harms of frequent social media use for adolescent health among a nationally representative sample of U.S. high school students. Findings might support multisectoral efforts to create safer digital environments for youths, including decision-making about social media policies, practices, and protections.

## Introduction

Social media, defined as “Internet-based channels that allow users to opportunistically interact and selectively self-present, either in real-time or asynchronously, with both broad and narrow audiences who derive value from user-generated content and the perception of interaction with others*,*” has become a pervasive presence in everyday life, including among youths (*1*). Recent data indicate that approximately 95% of high school–aged youths use a social media platform, with approximately one fifth reporting “almost constant” social media use (*2*). Associations between frequent social media use and poor mental health outcomes among adolescents, including depression (*3*) and suicide risk (*4*), are being increasingly documented. Social media use might also increase risk for electronic victimization and perpetration (*5*), which can be antecedents of poor mental health. Evidence suggests that certain youth populations might be more vulnerable than others to potential harms of social media use, such as female and lesbian, gay, bisexual, transgender, and queer or questioning adolescents, who are more likely to experience electronic victimization than male or heterosexual peers (*5*–*7*). However, youths might also benefit from social support and connection found online (*4*,*8*). Understanding potential risks and benefits of social media use is critical for preparing youths to safely engage in an increasingly digitalized world.

This report uses 2023 Youth Risk Behavior Survey (YRBS) data to build on extant literature by examining associations between frequent social media use and U.S. high school students’ experiences of bullying victimization, persistent feelings of sadness or hopelessness, and suicide risk. Understanding such patterns and relations might guide public health practitioners’ efforts to prevent violence and injury and promote mental health, in line with Healthy People 2030 objectives (https://health.gov/healthypeople). Findings from this report might also support multilevel decision-making about social media use and cross-sectoral initiatives (e.g., education, technology, and policy) to create safer digital environments for youths.

## Methods

### Data Source

This report includes data from the 2023 YRBS (N = 20,103), a cross-sectional, school-based survey conducted biennially since 1991. Each survey year, CDC collects data from a nationally representative sample of public and private school students in grades 9–12 in the 50 U.S. states and the District of Columbia. Additional information about YRBS sampling, data collection, response rates, and processing is available in the overview report of this supplement (*9*). The prevalence estimates for frequent social media use for the study population overall and stratified by sex, race and ethnicity, grade, and sexual identity are available at https://nccd.cdc.gov/youthonline/App/Default.aspx. The full YRBS questionnaire, data sets, and documentation are available at https://www.cdc.gov/yrbs/index.html. Institutional reviews boards at CDC and ICF, the survey contractor, approved the protocol for YRBS. Data collection was conducted consistent with applicable Federal law and CDC policy.*

### Measures

The primary exposure, frequency of social media use, was derived from the question, “How often do you use social media?” On the basis of response patterns, responses were dichotomized to reflect whether students used social media at least several times a day (frequent social media use [yes or no]) (Table 1). Six health behaviors or experiences were measured and dichotomized: bullying victimization (bullied at school or electronically bullied; past 12 months [yes or no]), mental health (persistent feelings of sadness or hopelessness; past 12 months [yes or no]), and suicide risk (seriously considered attempting suicide, made a suicide plan, or attempted suicide; past 12 months [yes or no]) (Table 2). The 2023 YRBS questionnaire defined bullying as “when one or more students tease, threaten, spread rumors about, hit, shove, or hurt another student over and over again. It is not bullying when two students of about the same strength or power argue or fight or tease each other in a friendly way.”

**TABLE 1:** Unweighted percentages for social media use item by response options — Youth Risk Behavior Survey, United States, 2023*

Response option	No. (%)
**How often do you use social media?**	
Less frequent social media use	3,331 (23.0)
1. I do not use social media	1,082 (7.6)
2. A few times a month	406 (2.9)
3. About once a week	231 (1.6)
4. A few times a week	708 (4.8)
5. About once a day	904 (6.1)
Frequent social media use	11,872 (77.0)
6. Several times a day	5,888 (40.1)
7. About once an hour	1,181 (7.4)
8. More than once an hour	4,803 (29.5)
Missing	4,900 (—)

* N = 20,103 respondents.

**TABLE 2:** Questions, response options, and analytic coding for frequency of social media use, bullying victimization, persistent feelings of sadness or hopelessness, and suicide risk among high school students — Youth Risk Behavior Survey, United States, 2023

Variable	Question	Response option	Analytic coding
Frequency of social media use	How often do you use social media*?	I do not use social media, a few times a month, about once a week, about once a day, several times a day, about once an hour, or more than once an hour	≥several times a day (frequent social media use) versus <several times a day (less frequent social media use)
Bullied at school	During the past 12 months, have you ever been bullied on school property?	Yes or no	Yes versus no
Electronically bullied	During the past 12 months, have you ever been electronically bullied? (Count being bullied through texting, Instagram, Facebook, or other social media.)	Yes or no	Yes versus no
Persistent feelings of sadness or hopelessness	During the past 12 months, did you ever feel so sad or hopeless almost every day for 2 weeks or more in a row that you stopped doing some usual activities?	Yes or no	Yes versus no
Seriously considered attempting suicide	During the past 12 months, did you ever seriously consider attempting suicide?	Yes or no	Yes versus no
Made a suicide plan	During the past 12 months, did you make a plan about how you would attempt suicide?	Yes or no	Yes versus no
Attempted suicide	During the past 12 months, how many times did you actually attempt suicide?	0 times, 1 time, 2 or 3 times, 4 or 5 times, or ≥6 times	≥1 time versus 0 times

* The 2023 National Youth Risk Behavior Survey questionnaire describes social media “such as Instagram, TikTok, Snapchat, and Twitter.”

Demographic variables included sex (female or male), race and ethnicity, age group (≤14, 15, 16, 17, or ≥18 years), and sexual identity (heterosexual [straight], lesbian or gay, bisexual, questioning [I am not sure about my sexual identity/questioning], or described identity in some other way [I describe my identity some other way]). In the 2023 YRBS, sexual identity and gender identity were measured separately; only sexual identity is included in this analysis. Race and ethnicity were coded as American Indian or Alaska Native (AI/AN), Asian, Black or African American (Black), Native Hawaiian or other Pacific Islander (NH/OPI), White, Hispanic or Latino (Hispanic), or multiracial (selected more than one racial category). (Persons of Hispanic or Latino origin might be of any race but are categorized as Hispanic; all racial groups are non-Hispanic).

### Analysis

Descriptive analyses examined point prevalence estimates and corresponding 95% CIs for frequent social media use in the overall sample and by demographic characteristics. Chi-square tests and pairwise *t*-tests were used to compare demographic group differences. Associations between frequent social media use and health behaviors and experiences (bullying victimization, persistent feelings of sadness or hopelessness, and suicide risk) were assessed in overall and separate logistic regression models stratified by sex or sexual identity, which generated prevalence ratios (PRs) and adjusted PRs (aPRs) for each health behavior and experience. All models were adjusted for demographic variables of race and ethnicity, age, sex, and sexual identity. If a model was stratified by a demographic characteristic, then the model was not adjusted for this characteristic. All prevalence estimates and measures of association used Taylor series linearization. Prevalence ratios were calculated using logistic regression with predicted marginals. Estimates were considered statistically significant if the aPR 95% CIs did not include 1.0 or p value was <0.05. All analyses were conducted in SAS-callable SUDAAN (version 11.0.3; RTI International) using sample weights to account for complex survey design and nonresponse.

## Results

Overall, 77.0% of U.S. high school students reported using social media at least several times a day (i.e., frequent social media use) (Table 3). Frequent social media use was more prevalent among female students compared with male students (81.8% versus 72.9%). Heterosexual students reported higher prevalence of frequent social media use than lesbian or gay students (79.2% versus 67.7%). Lesbian or gay students also reported lower prevalence of frequent social media use than students who identified as bisexual (82.2%), questioning (82.6%), or described their sexual identity in some other way (78.8%). AI/AN students had lower prevalence of frequent social media use (53.0%) than Asian, Black, White, Hispanic, or multiracial students.

**TABLE 3:** Prevalence of frequent social media use among high school students, overall and by selected demographic characteristics — Youth Risk Behavior Survey, United States, 2023*

Characteristic	Frequent social media use (n = 11,872)^†^	Chi-square test p value^¶^
	% (95% CI)^§^	
**Overall**	**77.0 (73.5–80.1)**	**—**
Sex**	—	0.0000
Female	81.8 (77.6–85.3)	—
Male	72.9 (69.8–75.8)	—
**Race and ethnicity^††^**	—	0.4503
American Indian or Alaska Native^§§^	53.0 (33.7–71.5)	—
Asian	75.8 (68.1–82.1)	—
Black or African American	78.7 (75.8–81.2)	—
Native Hawaiian or other Pacific Islander	75.8 (63.1–85.2)	—
White	76.7 (72.9–80.0)	—
Hispanic or Latino	78.0 (71.6–83.2)	—
Multiracial	76.3 (69.3–82.2)	—
**Age, yrs**	—	0.4937
≤14	74.5 (68.9–79.4)	—
15	76.1 (72.7–79.1)	—
16	77.0 (72.6–80.9)	—
17	79.1 (74.3–83.1)	—
≥18	77.1 (73.2–80.6)	—
**Sexual identity**	—	0.0587
Heterosexual (straight)^¶¶^	79.2 (77.2–81.1)	—
Lesbian or gay***	67.7 (57.8–76.3)	—
Bisexual	82.2 (79.3–84.9)	—
Questioning	82.6 (76.2–87.6)	—
Described identity in some other way	78.8 (70.7–85.2)	—

* N = 20,103 respondents. The total number of students answering each question varied. Data might be missing because 1) the question did not appear in that student’s questionnaire, 2) the student did not answer the question, or 3) the response was set to missing because of an out-of-range response or logical inconsistency. Percentages in each category are calculated on the known data. A total of 15,203 students responded to the social media item.

^†^ Unweighted.

^§^ Weighted.

^¶^ Chi-square tests were applied to examine the bivariate relations between demographic characteristics and frequency of social media use. Statistical significance is defined as p<0.05 for the chi-square test.

** Female students significantly differed from male students for prevalence of using of social media at least several times a day based on *t*-test with Taylor series linearization (p<0.05).

^††^ Persons of Hispanic or Latino origin might be of any race but are categorized as Hispanic; all racial groups are non-Hispanic.

^§§^ American Indian or Alaska Native students significantly differed from Asian, Black or African American, White, Hispanic or Latino, and multiracial students for prevalence of using social media at least several times a day based on *t*-test with Taylor series linearization (p<0.05).

^¶¶^ Heterosexual (straight) students significantly differed from lesbian or gay students for prevalence of using social media at least several times a day based on *t*-test with Taylor series linearization (p<0.05).

*** Lesbian or gay students significantly differed from bisexual and questioning students and students who described identity in some other way for prevalence of using social media at least several times a day based on *t*-test with Taylor series linearization (p<0.05).

Students who reported frequent social media use were more likely to be bullied at school and electronically bullied compared with less frequent social media users (Table 4). Frequent social media users also were more likely to report persistent feelings of sadness or hopelessness. Frequent social media use was associated with having seriously considered attempting suicide and having made a suicide plan.

**TABLE 4:** Prevalence estimates, unadjusted, and adjusted prevalence ratios for bullying victimization, mental health, and suicide risk among high school students, stratified by frequency of social media use — Youth Risk Behavior Survey, United States, 2023*

Health behavior and experience (past 12 months)	Frequent social media use		PR^†^ (95% CI)	aPR^§^ (95% CI)
	Yes	No		
	% (95%CI)	% (95%CI)		
**Bullying victimization**				
Bullied at school	19.9 (18.3–21.4)	19.0 (12.9–27.1)	1.05 (0.72–1.52)	1.31 (1.12–1.53)^¶^
Electronically bullied	17.0 (15.7–18.4)	15.9 (8.1–28.7)	1.07 (0.57–2.02)	1.54 (1.26–1.88)^¶^
**Mental health**				
Persistent feelings of sadness or hopelessness	42.6 (40.4–44.8)	31.9 (25.3–39.3)	1.33 (1.07–1.65)^¶^	1.35 (1.23–1.47)^¶^
**Suicide risk**				
Seriously considered attempting suicide	20.2 (18.8–21.8)	18.7 (12.8–26.6)	1.08 (0.75–1.55)	1.21 (1.06–1.37)^¶^
Made a suicide plan	16.6 (15.1–18.2)	17.5 (10.3–27.9)	0.95 (0.58–1.55)	1.16 (1.00–1.35)^¶^
Attempted suicide	9.5 (8.4–10.8)	9.5 (6.6–13.5)	1.00 (0.70–1.43)	1.11 (0.89–1.39)

**Abbreviations**: aPR = adjusted prevalence ratio; PR = prevalence ratio.

* N = 20,103 respondents. The total number of students answering each question varied. Data might be missing because 1) the question did not appear in that student’s questionnaire, 2) the student did not answer the question, or 3) the response was set to missing because of an out-of-range response or logical inconsistency. Percentages in each category are calculated on the known data. A total of 15,203 students responded to the social media item.

^†^ Logistic regression models estimated health behaviors and experiences between those who did and did not use social media at least several times a day.

^§^ Adjusted for age, race and ethnicity, sex, and sexual identity estimated health behaviors and experiences behaviors between those who did and did not use social media at least several times a day.

^¶^ Estimates were considered statistically significant if the 95% CIs did not include 1.0. Certain statistically significant aPRs have 95% CIs that include 1.0 because of rounding.

In sex-stratified analysis, female students who reported frequent social media use were more likely to experience bullying victimization at school and electronically compared with less frequent female social media users (Table 5). Female students who reported frequent social media use were also more likely to report persistent feelings of sadness or hopelessness and having seriously considered attempting suicide. Among male students, frequent social media users were more likely to experience bullying victimization electronically. Male students who frequently used social media also were more likely to report persistent feelings of sadness or hopelessness and having seriously considered attempting suicide.

**TABLE 5:** Prevalence estimates and unadjusted and adjusted prevalence ratios for bullying victimization, mental health, and suicide risk among high school students, by frequency of social media use and sex — Youth Risk Behavior Survey, United States, 2023*

Health behavior and experience (past 12 months)	Frequent social media use							
	Female				Male			
	Yes	No	PR^†^ (95% CI)	aPR^§^ (95% CI)	Yes	No	PR^¶^ (95% CI)	aPR** (95% CI)
	% (95% CI)	% (95% CI)			% (95% CI)	% (95% CI)		
**Bullying victimization**								
Bullied at school	23.0 (21.0–25.2)	19.5 (12.3–29.4)	1.18 (0.76–1.84)	1.54 (1.19–1.98)^††^	16.6 (14.9–18.4)	18.3 (12.3–26.3)	0.90 (0.61–1.35)	1.17 (0.93–1.47)
Electronically bullied	21.5 (19.7–23.5)	20.0 (10.0–36.1)	1.08 (0.57–2.04)	1.66 (1.31–2.09)^††^	12.2 (10.7–13.8)	13.0 (6.6–24.0)	0.94 (0.48–1.85)	1.48 (1.09–2.00)^††^
**Mental health**								
Persistent feelings of sadness or hopelessness	55.1 (52.3–57.9)	44.4 (37.0–52.1)	1.24 (1.05–1.46)	1.32 (1.20–1.46)^††^	29.5 (27.8–31.3)	24.0 (17.8–31.5)	1.23 (0.92–1.64)	1.41 (1.20–1.67)^††^
**Suicide risk**								
Seriously considered attempting suicide	26.4 (24.4–28.5)	26.8 (18.4–37.2)	0.98 (0.70–1.39)	1.18 (1.00–1.39)^††^	13.9 (12.2–15.8)	13.6 (9.10–19.8)	1.02 (0.69–1.52)	1.25 (1.04–1.49)^††^
Made a suicide plan	21.5 (19.5–23.7)	22.7 (13.4–35.8)	0.95 (0.58–1.54)	1.24 (0.96–1.59)	11.5 (10.3–12.8)	14.1 (8.3–23.0)	0.81 (0.49–1.34)	1.10 (0.90–1.33)
Attempted suicide	12.5 (11.0–14.2)	13.5 (9.0–19.8)	0.92 (0.63–1.36)	1.16 (0.85–1.57)	6.3 (5.1–7.7)	6.6 (4.6–9.2)	0.96 (0.68–1.35)	1.03 (0.77–1.39)

**Abbreviations**: PR = prevalence ratio; aPR = adjusted prevalence ratio.

* N = 20,103 respondents. The total number of students answering each question varied. Data might be missing because 1) the question did not appear in that student’s questionnaire, 2) the student did not answer the question, or 3) the response was set to missing because of an out-of-range response or logical inconsistency. Percentages in each category are calculated on the known data. A total of 15,203 students responded to the social media question.

^†^ Logistic regression models estimated health behaviors and experiences between those who did and did not use social media at least several times a day, among female students.

^§^ Adjusted for age, race and ethnicity, and sexual identity estimated health behaviors and experiences between those who did and did not use social media at least several times a day, among female students.

^¶^ Logistic regression models estimated health behaviors and experiences between those who did and did not use social media at least several times a day, among male students.

** Adjusted for age, race and ethnicity, and sexual identity estimated health behaviors and experiences between those who did and did not use social media at least several times a day, among male students.

^††^ Estimates were considered statistically significant if the 95% CIs did not include 1.0. Certain statistically significant aPRs have 95% CIs that include 1.0 because of rounding.

In sexual identity–stratified analyses, students who identified as lesbian or gay, bisexual, questioning, or described their identity in some other way (LGBQ+) and who reported frequent social media use were more likely to experience bullying victimization electronically and persistent feelings of sadness or hopelessness than less frequent LGBQ+ social media users (Table 6). Among heterosexual students, both unadjusted and adjusted analyses found that those who were frequent social media users were more likely than less frequent social media users to experience all observed health behaviors and experiences except for attempted suicide.

**TABLE 6:** Prevalence estimates and unadjusted and adjusted prevalence ratios for bullying victimization, mental health, and suicide risk among high school students, by frequency of social media use and sexual identity — Youth Risk Behavior Survey, United States, 2023*

Health behavior and experience (past 12 months)	Frequent social media use							
	LGBQ+				Heterosexual (straight)			
	Yes	No	PR^†^ (95%CI)	aPR^§^ (95%CI)	Yes	No	PR^¶^ (95%CI)	aPR** (95%CI)
	% (95%CI)	% (95%CI)			% (95%CI)	% (95%CI)		
**Bullying victimization**								
Bullied at school	29.4 (25.5–33.5)	27.2 (21.8–33.5)	1.08 (0.86–1.35)	1.25 (0.96–1.63)	16.8 (15.4–18.4)	12.2 (10.3–14.5)	1.37 (1.15–1.65)^††^	1.33 (1.12–1.58)^††^
Electronically bullied	25.5 (21.9–29.4)	18.6 (14.3–23.7)	1.37 (1.03–1.82)^††^	1.50 (1.14–1.99)^††^	14.4 (13.2–15.7)	8.6 (6.8–10.8)	1.67 (1.32–2.12)^††^	1.55 (1.24–1.94)^††^
**Mental health**								
Persistent feelings of sadness or hopelessness	68.6 (65.5–71.6)	52.9 (45.5–60.3)	1.30 (1.13–1.49)^††^	1.23 (1.06–1.44)^††^	34.4 (32.4–36.4)	22.3 (19.3–25.5)	1.54 (1.36–1.76)^††^	1.42 (1.27–1.60)^††^
**Suicide risk**								
Seriously considered attempting suicide	40.4 (37.3–43.7)	35.3 (30.5–40.4)	1.15 (0.99–1.32)	1.09 (0.93–1.28)	13.9 (12.7–15.1)	10.0 (7.9–12.5)	1.39 (1.12–1.73)^††^	1.33 (1.08–1.64)^††^
Made a suicide plan	32.4 (29.3–35.6)	31.1 (25.6–37.2)	1.04 (0.86–1.25)	1.00 (0.81–1.22)	11.7 (10.6–13.0)	8.2 (6.4–10.4)	1.43 (1.12–1.83)^††^	1.37 (1.07–1.75)^††^
Attempted suicide	19.4 (16.7–22.4)	18.7 (14.6–23.7)	1.04 (0.77–1.40)	1.00 (0.71–1.41)	6.3 (5.5–7.2)	5.0 (3.2–7.8)	1.25 (0.79–1.96)	1.24 (0.88–1.76)

**Abbreviations**: aPR = adjusted prevalence ratio LGBQ+ = lesbian or gay, bisexual, questioning, or described identity in some other way; PR = prevalence ratio.

* N = 20,103 respondents. The total number of students answering each question varied. Data might be missing because 1) the question did not appear in that student’s questionnaire, 2) the student did not answer the question, or 3) the response was set to missing because of an out-of-range response or logical inconsistency. Percentages in each category are calculated on the known data. A total of 15,203 students responded to the social media question.

^†^ Logistic regression models estimated health behaviors and experiences between those who did and did not use social media at least several times a day, among LGBQ+ students.

^§^ Adjusted for age, race and ethnicity, and sex estimated health behaviors and experiences between those who did and did not use social media at least several times a day, among LGBQ+ students.

^¶^ Logistic models estimated health behaviors and experiences between those who did and did not use social media at least several times a day, among heterosexual students.

** Adjusted for age, race and ethnicity, and sex estimated health behaviors and experiences between those who did and did not use social media at least several times a day, among heterosexual students.

^††^ Estimates were considered statistically significant if the 95% CIs did not include 1.0. Certain statistically significant aPRs have 95% CIs that include 1.0 because of rounding.

## Discussion

This report provides the first national prevalence estimate of social media use from a representative sample of U.S. high school students. Findings suggest that most high school students use social media, and that a substantial majority (77.0%) use social media frequently (i.e., at least several times a day) (Table 1). Frequent social media use was largely consistent across demographic characteristics, highlighting the widespread presence of social media during adolescence. Therefore, it remains critical to strengthen collective understanding of potential risks and benefits of social media use for adolescent health and development, and in turn, understand how to create safe digital environments and help youths develop and maintain healthy digital practices that minimize harm (*1*).

Certain differences in students’ social media use by sex, racial and ethnic identity, and sexual identity were observed. In alignment with previous literature, female students reported higher prevalence of frequent social media use than male students (*6*). AI/AN students reported less frequent social media use compared with those of other racial and ethnic identities, which might reflect differences in broadband Internet access between rural and tribal communities and other communities in the United States (*10*). Lesbian and gay students reported less frequent social media use compared with peers of other sexual identities. This finding contrasts with certain previous literature indicating that lesbian, gay, and bisexual youths might spend more time engaging with identity-affirming communities online, often through social media (*8*). Further research is needed to understand nuances of social media use among youths and the impact of social media on health and well-being for different youth populations.

Consistent with previous research, frequent social media users were more likely to experience bullying victimization (*5*). Previous research has demonstrated evidence of overlap between in-person and electronic bullying contexts, with perpetrators of in-person bullying more likely to perpetrate electronic bullying, and victims of in-person bullying more likely to experience electronic bullying victimization and engage in bullying perpetration (*11*). Such interplay between in-person and electronic bullying environments might explain the finding of higher prevalence of bullying at school among frequent versus less frequent social media users. However, additional research is needed to better understand this phenomenon and the compounding impact of bullying victimization across multiple contexts on adolescents’ short- and long-term thriving (*11*).

Associations between frequent social media use and bullying victimization differed by sex and sexual identity. Female students who reported frequent social media use were more susceptible to bullying victimization compared with less frequent female social media users. This might reflect the types of victimization (e.g., relational and psychological) commonly experienced by adolescent girls (*12*), which are suited to digital environments that reduce barriers to conflict (e.g., anonymity and proximity). Among LGBQ+ students, frequent social media users were more likely to experience electronic bullying victimization than less frequent social media users yet demonstrated no significant differences in bullying victimization at school. In contrast, heterosexual students who used social media frequently were more likely to experience both types of bullying victimization compared with heterosexual students who used social media less often. One possible explanation is that LGBQ+ students who use social media frequently have greater exposure to online discrimination or stigma-based bullying victimization beyond school networks (*7*,*8*). Therefore, frequent and less frequent social media users could share similar experiences of bullying in at-school networks but different experiences electronically. Further research is needed to understand variations in at-school and electronic networks for youths of different identities and how overlap between at-school and electronic networks might influence bullying victimization.

In alignment with existing research, findings in this report support associations between adolescent social media use and mental health; specifically, frequent social media users were more likely to report persistent feelings of sadness or hopelessness (*3*). Adjusted stratified analyses demonstrated consistent associations across groups, conveying a shared risk for poor mental health among students who are frequent social media users. However, literature also suggests that certain groups are more vulnerable to the potential negative mental health impacts of social media than others (e.g., adolescent girls) (*6*). In this study, approximately half of female students and one third of LGBQ+ students who frequently used social media reported persistent feelings of sadness or hopelessness, respectively. Findings warrant more rigorous analyses inclusive of multiple mental health indicators to better understand differential impact of frequent social media use by sex, sexual identity, and other key demographic characteristics.

Overall, frequent social media users were more likely to report having seriously considered attempting suicide and having made a suicide plan. No significant differences in reports of attempted suicide by frequency of social media use were observed, perhaps because of the rarity of this behavior in the sample. These findings mirror broader inconsistencies in the literature (*4**,**13*). Certain researchers posit that the relation between social media use and suicide risk is more complex and indirect than a dose-response phenomenon (*4*,*13*). For example, differences in how adolescents are exposed to suicide-related content have been demonstrated to influence suicide risk. More interactive and proximate exposures via online discussion forums or suicide clusters might increase risk compared with passive media consumption (*4*,*14*). In addition, analyses did not describe indirect pathways (e.g., through online victimization or reduced sleep quality) through which frequent social media use might influence mental health and suicide risk, or protective factors (e.g., connectedness to others) that might buffer the negative impacts of frequent social media use on mental health and suicide risk (*4*). Because of persistent concerns about the impact of social media on youth mental health (*1*), additional research is needed to better understand how such pathways might moderate the relation between frequent social media use and suicide risk.

In stratified analyses, associations between frequent social media use and suicide risk diminished, except for heterosexual students. This group might be a factor in the small, significant association between social media use and making a suicide plan observed in the overall sample. Findings suggest that heterosexual students might be more vulnerable to negative impacts of social media on suicide risk. This is surprising because of high prevalence of suicide risk among LGBQ+ students in the sample, but also suggests that social media might not be the most influential factor of suicide risk for LGBQ+ students. Emerging literature has found that social media can be protective for youths who identify as LGBTQ+ by connecting them with affirming communities, support networks, and resources online (*8*) and might even reduce suicide risk for certain youths (*4*). More research is needed to understand potential protective effects of positive connections made through safe and supportive social media environments and their associations with bullying victimization, suicide risk, and mental health.

## Limitations

General limitations of the YRBS are available in the overview report of this supplement (*9*). Findings in this report are subject to at least six additional limitations. First, YRBS data are cross-sectional; causality and directionality of associations between frequent social media use and health behaviors and experiences cannot be established. Second, YRBS examples of social media were not exhaustive; students might engage in other online platforms that were not considered in responses to the social media item. Third, differences between social media nonusers and infrequent users might be masked. Responses to the social media item were dichotomized to ensure sufficient statistical power, and respondents who selected “I do not use social media” were grouped with less frequent social media users (Table 1). Fourth, to maintain consistency in recall period across health behaviors and experiences, analyses only included one mental health indicator; students reporting on other indicators of poor mental health might have been missed. Fifth, sexual identities were dichotomized into two broad categories in stratified analysis because of sample size limitations. Because of significantly lower prevalence of frequent social media use among lesbian and gay students, combining them with students of other sexual identities might have hidden possible stronger effects or differences for other identities. Finally, with the availability of social media, bullying victimization at school can occur in person or electronically; similarly, electronic bullying can happen at school or elsewhere. Therefore, the two bullying victimization measures (i.e., at school and electronically) might not be mutually exclusive because these two pathways of bullying might overlap.

## Future Directions

Findings from this study highlight key areas for future research and practice regarding youth social media use and related health behaviors and experiences. This study identified important differences in frequent social media use and its impact on bullying victimization, persistent feelings of sadness and hopelessness, and suicide risk by sex and sexual identity; however, consensus is lacking about how best to measure social media use (*3*,*4*). Future research that identifies how different social media measures (e.g., frequency of use, passive versus active use, and addiction to use) might differentially describe social media and related health outcomes is important to further understanding of potential risks and benefits of youth social media use. In addition, these findings warrant additional exploration of the differential association of social media use with bullying, mental health, and suicide risk by racial and ethnic identity of youths along with more detailed analyses of differences by sexual identity and gender identity. Investigating such associations among frequent social media users might increase understanding about which students are more vulnerable to the negative impacts of frequent social media use. Future research exploring the pathways through which social media use might lead to poor mental health and suicide risk, including through cyberbullying and victimization, also is needed.

Improved understanding of youths’ social media use and related health outcomes can strengthen cross-sectoral endeavors to create safer digital environments, such as consumer safety policies, media literacy education and standards, and platform-based protections for youths online (*1*). This understanding might also help empower youths and families to make informed decisions about social media use and online behaviors that reduce risk for negative health outcomes, including bullying victimization, poor mental health, and suicide (*1*). School-based interventions that address bullying and suicide prevention have been proven to be effective (*15*,*16*). Strengthening youths’ health-enhancing skills, creating protective environments, and promoting connections to positive adults and peers through programs such as What Works in Schools (https://www.cdc.gov/healthyyouth/whatworks/index.htm) can help reduce risk for multiple forms of violence and suicide (*17*). CDC’s Community Violence Prevention Resource for Action (https://www.cdc.gov/violence-prevention/media/pdf/resources-for-action/CV-Prevention-Resource-for-Action_508.pdf) and Suicide Prevention Resource for Action (https://www.cdc.gov/suicide/resources/prevention.html) contain strategies based on the best available evidence to reduce community violence, including youth violence and bullying, and suicide. StopBullying.gov (https://www.stopbullying.gov/prevention/how-to-prevent-bullying) provides steps that schools, youths, and their families can take to prevent bullying, including setting clear behavioral expectations and promoting empathy, self-awareness, and self-regulation skills. The U.S. Surgeon General’s Advisory on Social Media and Youth Mental Health (https://www.hhs.gov/surgeongeneral/priorities/youth-mental-health/social-media/index.html#action) and American Academy of Pediatrics’ Center of Excellence on Social Media and Youth Mental Health (https://www.aap.org/en/patient-care/media-and-children/center-of-excellence-on-social-media-and-youth-mental-health) provide recommendations on ways youths and families can reduce risk for harm from social media use (e.g., developing family media plans to promote healthy social media use). More research is needed to rigorously test and evaluate interventions that incorporate evidence-based prevention strategies among youths who use social media, particularly those at increased risk for harms associated with frequent social media use.

## Conclusion

Overall, approximately three fourths of U.S. high school students reported using social media at least several times a day. Frequent social media use among students was associated with higher prevalence of bullying victimization at school and electronically, persistent feelings of sadness and hopelessness, having seriously considered attempting suicide, and having made a suicide plan. Associations between frequent social media use and these health behaviors and experiences differed by sex and sexual identity. Although additional research is needed to understand precisely how social media use differentially affects adolescent risk for bullying victimization, poor mental health, and suicide, existing evidence-based prevention strategies can be used by families, schools, and communities to promote adolescent mental health and prevent injury and violence.

## Footnotes

* 45 C.F.R. part 46.114; 21 C.F.R. part 56.114.

## References

[1] Office of the Surgeon General. Social media and youth mental health: the U.S. Surgeon General’s advisory. Washington DC: US Department of Health and Human Services, Office of the Surgeon General; 2023. https://www.hhs.gov/sites/default/files/sg-youth-mental-health-social-media-advisory.pdf

[2] Anderson M, Faverio M, Gottfried J. Teens, social media and technology 2023. Washington, DC: Pew Research Center; 2023. https://www.pewresearch.org/internet/2023/12/11/teens-social-media-and-technology-2023

[3] Keles B, McCrae N, Grealish A. A systematic review: the influence of social media on depression, anxiety and psychological distress in adolescents. Int J Adolesc Youth 2020;25:79–93. 10.1080/02673843.2019.1590851

[4] Macrynikola N, Auad E, Menjivar J, Miranda R. Does social media use confer suicide risk? A systematic review of the evidence. Comput Hum Behav Rep 2021;3:100094. 10.1016/j.chbr.2021.100094

[5] Craig W, Boniel-Nissim M, King N, Social media use and cyber-bullying: a cross-national analysis of young people in 42 countries. J Adolesc Health 2020;66(6S):S100–8. 10.1016/j.jadohealth.2020.03.006 32446603

[6] Twenge JM, Haidt J, Lozano J, Cummins KM. Specification curve analysis shows that social media use is linked to poor mental health, especially among girls. Acta Psychol (Amst) 2022;224:103512. 10.1016/j.actpsy.2022.103512 35101738

[7] Abreu RL, Kenny MC. Cyberbullying and LGBTQ youth: a systematic literature review and recommendations for prevention and intervention. J Child Adolesc Trauma 2017;11:81–97. 10.1007/s40653-017-0175-7 32318140 PMC7163911

[8] Berger MN, Taba M, Marino JL, Lim MSC, Skinner SR. Social media use and health and well-being of lesbian, gay, bisexual, transgender, and queer youth: systematic review. J Med Internet Res 2022;24:e38449. 10.2196/38449 36129741 PMC9536523

[9] Brener ND, Mpofu JJ, Krause KH, Overview and methods for the Youth Risk Behavior Surveillance System—United States, 2023. In: Youth Risk Behavior Surveillance—United States, 2023. MMWR Suppl 2024;73(No. Suppl-4):1–12. 10.15585/mmwr.su7304a1 PMC11559678 39378301

[10] Early J, Hernandez A. Digital disenfranchisement and COVID-19: broadband Internet access as a social determinant of health. Health Promot Pract 2021;22:605–10. 10.1177/15248399211014490 33955266

[11] Lazuras L, Barkoukis V, Tsorbatzoudis T. Face-to-face bullying and cyberbullying in adolescents: trans-contextual effects and role overlap. Technol Soc 2017;48:97–104. 10.1016/j.techsoc.2016.12.001

[12] National Academies of Sciences, Engineering, and Medicine. Preventing bullying through science, policy, and practice. Washington DC: National Academies Press; 2016. 27748087

[13] Nesi J, Burke TA, Bettis AH, Social media use and self-injurious thoughts and behaviors: a systematic review and meta-analysis. Clin Psychol Rev 2021;87:102038. 10.1016/j.cpr.2021.102038 34034038 PMC8243901

[14] Swedo EA, Beauregard JL, de Fijter S, Associations between social media and suicidal behaviors during a youth suicide cluster in Ohio. J Adolesc Health 2021;68:308–16. 10.1016/j.jadohealth.2020.05.049 32646827 PMC8366066

[15] Community Preventive Services Task Force. Violence prevention: school-based anti-bullying interventions. Washington, DC: US Department of Health and Human Services, Community Preventive Services Task Force; 2022. https://www.thecommunityguide.org/findings/violence-prevention-school-based-anti-bullying-interventions.html

[16] Community Preventive Services Task Force. Mental health: multi-tiered trauma-informed school programs to improve mental health among youth. Washington, DC: US Department of Health and Human Services, Community Preventive Services Task Force; 2023. https://www.thecommunityguide.org/findings/mental-health-multi-tiered-trauma-informed-school-programs-improve-mental-health-among-youth.html

[17] Wilkins NJ, Rasberry C, Liddon N, Addressing HIV/sexually transmitted diseases and pregnancy prevention through schools: an approach for strengthening education, health services, and school environments that promote adolescent sexual health and well-being. J Adolesc Health 2022;70:540–9. 10.1016/j.jadohealth.2021.05.017 35305791 PMC9260911

